# The effect of sleep on male reproductive system: A protocol for systematic review and meta analysis

**DOI:** 10.1097/MD.0000000000022595

**Published:** 2020-10-02

**Authors:** Xiang Gao, Xiping Chen, Wei Huang, Shengyan Tan, Xinglong Liu, Xiaodan Zhang

**Affiliations:** Department of prescription, School of basic Medicine, Chengdu University of traditional Chinese Medicine, Chengdu.

**Keywords:** reproductive system, semen analyses, sleep, systematic review

## Abstract

Background: the purpose of this study was to evaluate the effects of sleep deprivation on semen analyses, hormone levels and testicular histopathology in men.

Methods: this review will be included in a qualified case-control study. The search strategy will be implemented in PubMed, Embase, Web of science, Cochrane library, China National knowledge Infrastructure database, Wanfang Database, and the Cochrane library. We will solicit both English and Chinese case-control studies published from its beginning to July 31, 2020. The 2 examiners will independently screen, select research, extract data and evaluate quality. We use Revman5.3 software to generate funnel map, heterogeneity assessment, data analysis, subgroup analysis and sensitivity analysis.

Results: in the current meta-analysis, we will provide some more practical and targeted results for the study of the effects of sleep on the male reproductive system, and sum up the main limitations of previous studies.

Conclusion: this study will provide new evidence for the effect of sleep on male reproductive system.

## 1 Introduction

Male infertility is considered to be a public-health-epidemic.[^1^,^2^] The World Health Organization (WHO)estimates that 8% to 12% of couples worldwide face fertility problems, while male factors cause 50% of the problems.^[3]^ There are many factors causing male infertility, such as environmental pollution, mental stress, smoking, alcoholism, bad lifestyle and so on.[^4^,^5^,^6^,^7^] in recent years, studies have shown that bad living habits can lead to a decline in semen analyses (SA).^[8]^ Therefore, people pay more and more attention to sleep. Sleep is of great significance to human health, which can affect people's nervous, endocrine, immune and cardiovascular system functions. Nevertheless, sleep management is still challenging and has not been fully understood. Therefore, we will conduct a systematic review and meta-analysis to determine the effects of sleep on SA and hormone levels, and to provide further evidence for health service providers and patients. The purpose of this study is to conduct a systematic review and meta-analysis to determine the effects of sleep on the male reproductive system (MRS).

## 2 Method

This protocol has been registered on the Open Science Framework (OSF) platform (Registration Number: DOI 10.17605/OSF.IO/3W5AU). We will strictly implement the network meta-analysis of systematic evaluation and meta-analysis reports in compliance with the requirements of the preferred reports. Ethical approval is not necessary for the study.

### 2.1 Inclusion criteria for study selection

#### 2.1.1 Study types

Case-control studies related to the effects of sleep on the MRS will be included in our evaluation. These studies provided available genotypic frequencies in the case of the group and the control group to estimate the ratio of and its 95% network connection confidence interval. The language, publication date or status of the study will not be subject to any restrictions.

#### 2.1.2 Participant types

Male participants affected by sleep will be included in the meta-analysis. The control subjects were defined as healthy subjects who slept normally. There is no restriction on age or country.

#### 2.1.3 Outcome

SA, containing sperm motility parameters and sperm count, hormone levels, including testosterone levels, corticosterone levels, progesterone levels, testicular pathological changes.

#### 2.1.4 Exclusion criteria

Repetitive reports, conference summaries, case reports, review papers, or animal studies, and so on.

#### 2.1.5 Search strategy

We will search all the qualified literatures in the China knowledge Network database, database of PubMed, Embase and Web of science. Search time range from the beginning to July 31, 2020 published all Chinese and English literature. In addition, considering unpublished or ongoing trials, or trials that have been completed but ready to be published, we will conduct targeted searches of grey literature on the clinical trial website and the China Clinical Trials Registry. At the same time, the reference list of previous clinical studies and reviews is used as a supplementary source. Take the retrieval of Pubmed database as an example, the retrieval strategy is shown in Table 1.

**Table 1:**
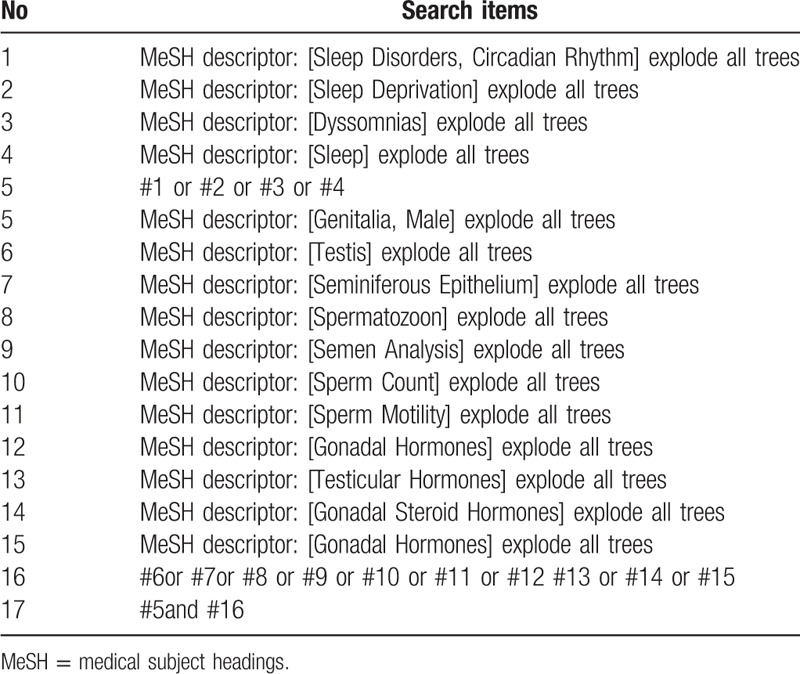
Search strategy used in PubMed database.

### 2.2 Data collection and analysis

#### 2.2.1 Research selections

**Figure 1:**
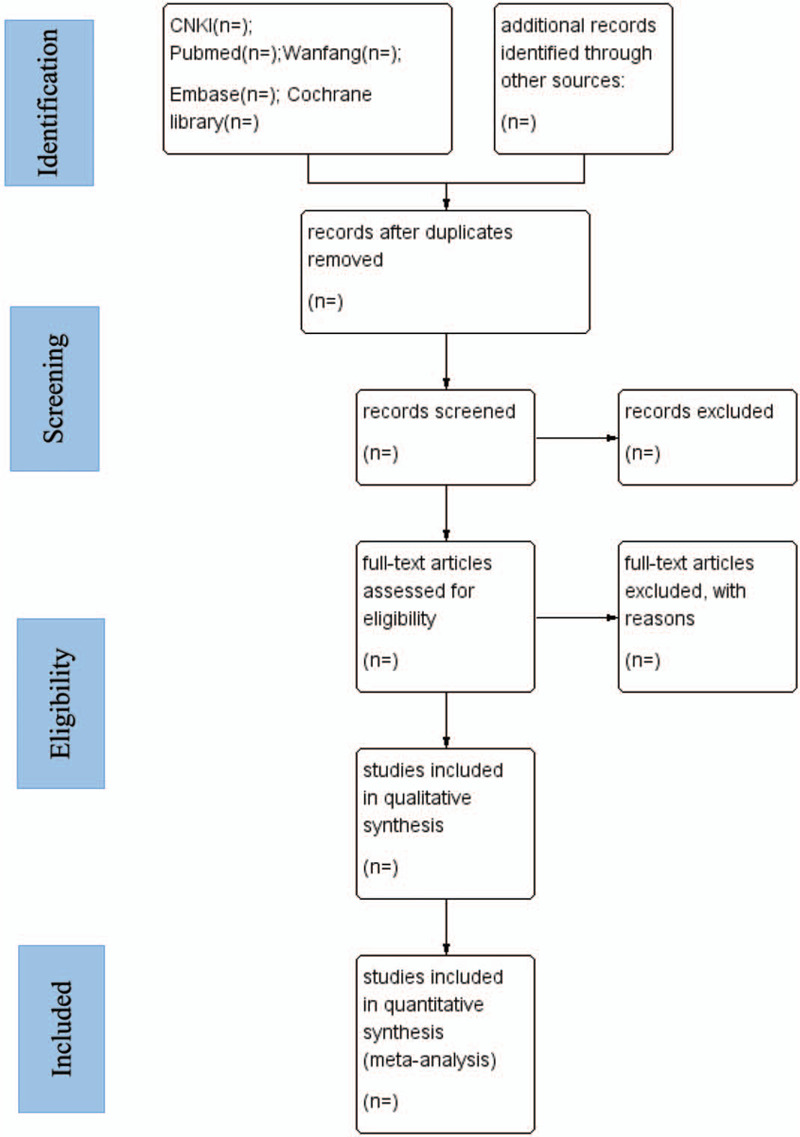
Flow chart of study selection.

The 2 examiners (LXL and YQB) will independently conduct a literature search and select titles and abstracts according to the established inclusion and exclusion criteria. Then read the full text as needed for further evaluation. In the process of searching and evaluating the literature, any differences will be discussed and resolved with the third-party author (ZXD). For uncertain data, we will get in touch with the corresponding first author to determine whether this literature is included. Details of the process of the research are shown in the following chart (Fig. 1).

#### 2.2.2 Extraction of data and information

Detailed data and information extraction tables are as follows: basic information (first author, year of publication, country, year of study); year of study, characteristics of participants, sample size, race, age, results. When Shengyan Tan and others deal with the lost data, when the literature data is lost or unclear, it will be solved through discussion in the group and contacting the corresponding authors. the specific method can be obtained by contacting the corresponding author by e-mail. If the problem cannot be solved, a sensitivity analysis is conducted to determine the influence of the contained study in the whole outcome assessment.

#### 2.2.3 Heterogeneity assessment

We will use *I* ^2^ statistics to quantify statistical heterogeneity to assess the heterogeneity included in studies. The degree of heterogeneity is according to: I^2^ = 0% ∼ 40%, which may not be significant, *I* ^*2*^ = 30% ∼ 60%, which can indicate moderate heterogeneity; *I* ^2^ = 50% ∼ 90%, which can indicate considerable heterogeneity; *I* ^2^ = 75% ∼ 100%, considerable heterogeneity.

#### 2.2.4 Evaluation of studying quality

The physiotherapy evidence database scale^[9]^ will be utilized by 2 independent evaluators (LXL) to evaluate 11 quality areas of each included literature. The maximum score for each item is 10 (standard 1 does not score), including random allocation, hidden allocation, appropriate animal model, blind evaluation results, sample size calculation, and compliance with animal welfare regulations. Any differences will be resolved through discussion and negotiation with Xiping Chen.

#### 2.2.5 Assessment of reporting deviations

If there are more than 10 studies, funnel charts will be used to assess the potential for biases in small studies. If necessary, we will utilize Egger regression test and Begger correlation test to evaluate the publication bias.

#### 2.2.6 Data synthesis

All analyses will be carried out using Revman 5.3 software. The outcome indicators reported in the above study will be meta-analyzed. In this meta-analysis, we will use a stochastic model. If the *I* ^*2*^ test indicates unacceptable heterogeneity (*I* ^2^ > 50%), we will conduct sensitivity analysis and subgroup analysis. If meta-analysis is not possible, the data will be carried out through a descriptive summary report. The probability value *P* < .05 is considered to be statistically significant.

#### 2.2.7 Dubgroup analysis

If there is any significant heterogeneity among the results, we will conduct a subgroup analysis: gene expression, study quality, sperm morphology.

#### 2.2.8 Sensitivity analysis

We will utilize sensitivity analysis to delete studies that do not conform to the HWE to assess the robustness and reliability of the summary results.

#### 2.2.9 Quality of evidence

We will use recommendation level, evaluation level, development level and evaluation level to assess the quality of these results. The results will be compartmentalized into 4 categories: very low, low, medium or high.^[10]^

## 3 Discuss

Insomnia is a wide range of sleep disorders caused by life events, or from a variety of pathophysiological causes, such as the effects of drugs, chronic diseases, and mental illness. Although there have been a large number of reports on the effects of sleep on the MRS. There are also many related animal studies, and the mechanism behind it is relatively well understood[^11^,^12^] High-quality evidence about the effects of sleep on the MRS is still lacking. However, the impact of sleep on the MRS is still unintelligible. We hope to find more practical and targeted results for determining the effects of sleep on the MRS in the recent systematic review and meta-analysis.

The advantages of this system review include: in order to avoid deviation, we will search the comprehensive literature as much as possible, including Chinese and English databases, and we will strictly evaluate the quality and extract the data. The deficiency of this review is that we only search the most commonly used medical databases, which may deviate the results.

## Author contributions

Shengyan Tan: project administration.

Xiang Gao: writing and drafting a plan.

Xiaodan Zhang and Wei huang: revising the protocol.

Xinglong Liu: research selection and data extraction.

Xiping Chen: resolving any differences.

**Data curation:** Xinglong Liu.

**Project administration:** Shengyan Tan.

**Software:** Wei Huang.

**Supervision:** Xiaodan Zhang.

**Writing – original draft:** Xiang Gao.

**Writing – review & editing:** Xiping Chen.

## References

[1] Vander Borght M Wyns C Fertility and infertility: definition and epidemiology. Clin Biochem 2018;62:2–10. 29555319 10.1016/j.clinbiochem.2018.03.012

[2] Tournaye H Krausz C Oates RD Novel concepts in the aetiology of male reproductive impairment. Lancet Diabetes Endocrinol 2017;5:544–53. 27395771 10.1016/S2213-8587(16)30040-7

[3] Agarwal A Mulgund A Hamada A A unique view on male infertility around the globe. Reprod Biol Endocrinol 2015;13:37. 25928197 10.1186/s12958-015-0032-1 PMC4424520

[4] Zilberlicht A Wiener-Megnazi Z Sheinfeld Y Habits of cell phone usage and sperm quality - does it warrant attention? Reprod Biomed Online 2015;31:421–6. 26206279 10.1016/j.rbmo.2015.06.006

[5] Vested A Ramlau-Hansen CH Olsen SF Associations of in utero exposure to perfluorinated alkyl acids with human semen quality and reproductive hormones in adult men. Environ Health Perspect 2013;121:453–8. 23360585 10.1289/ehp.1205118 PMC3620740

[6] Ricci E Viganò P Cipriani S Coffee and caffeine intake and male infertility: a systematic review. Nutr J 2017;16:37. 28646871 10.1186/s12937-017-0257-2 PMC5482951

[7] Skakkebaek NE Rajpert-De Meyts E Buck Louis GM Male reproductive disorders and fertility trends: influences of environment and genetic susceptibility. Physiol Rev 2016;96:55–97. 26582516 10.1152/physrev.00017.2015 PMC4698396

[8] Nassan FL Chavarro JE Tanrikut C Diet and men's fertility: does diet affect sperm quality? Fertil Steril 2018;110:570–7. 30196939 10.1016/j.fertnstert.2018.05.025

[9] Moher D Shamseer L Clarke M Preferred reporting items forsystematic review and meta-analysis protocols (PRISMA-P) 2015statement. Syst Rev 2015;4:1. 25554246 10.1186/2046-4053-4-1 PMC4320440

[10] Balshem H Helfand M Schünemann HJ GRADE guidelines: 3. Rating the quality of evidence. J Clin Epidemiol 2011;64:401–6. 21208779 10.1016/j.jclinepi.2010.07.015

[11] Alvarenga TA Hirotsu C Mazaro-Costa R Tufik S Andersen ML Impairment of male reproductive function after sleep deprivation. Fertil Steril. 2015; 103:1355-62.e1. Liu PY. A Clinical Perspective of Sleep and Andrological Health: Assessment, Treatment Considerations, and Future Research. J Clin Endocrinol Metab. 2019; 104:4398-4417. 10.1016/j.fertnstert.2015.02.002 25747127

[12] Choi JH Lee SH Bae JH Effect of sleep deprivation on the male reproductive system in rats. J Korean Med Sci 2016;31:1624–30. 27550492 10.3346/jkms.2016.31.10.1624 PMC4999406

